# Indoor Tanning within UK Young Adults: An Extended Theory of Planned Behaviour Approach

**DOI:** 10.5402/2013/394613

**Published:** 2012-09-17

**Authors:** Lorna J. Dodd, Mark J. Forshaw, Stella Williams

**Affiliations:** ^1^Department of Psychology & Counselling, Newman University College, Genners Lane, Bartley Green, Birmingham B32 3NT, UK; ^2^Centre for Health Psychology, Staffordshire University, Stoke-on-Trent ST4 2DE, UK

## Abstract

The indoor tanning industry poses a long-term public health risk. Despite the adverse health effects, indoor tanning seems to be gaining considerable popularity. The study examined indoor tanning intentions and behaviour within UK young adults using an extended theory of planned behaviour model, which included variables on “appearance reasons to tan,” “perceived susceptibility to damaging appearance,” “perceived susceptibility to health consequences,” and “tanning knowledge.” The model was successful in predicting indoor tanning intentions and behaviour (explained 17% and 71%, resp.). An interesting outcome was the magnitude of the variable “appearance reasons to tan.” A current tanned appearance therefore seemed to outweigh any adverse future appearance or health consequences caused by indoor tanning. Appearance-focused interventions to reduce such behaviour may now prove to be efficacious within a UK sample.

## 1. Introduction

The two main types of skin cancer linked to ultraviolet (UV) radiation are nonmelanoma and malignant melanoma [1]. The incidence and prevalence of both these skin cancers are increasing globally each year, with 2-3 million and 132,000 cases reported yearly, respectively [2]. UV radiation is emitted from both the sun and artificial tanning devices, the latter of which has been referred to as sunlamps, sunbeds, and tanning salons, henceforth referred to as indoor tanning—IT [3, 4]. Such exposure is also associated with premature aging, cataracts, and immune suppression [3, 4].

In climates such as Australia and the United States of America (USA), reports have shown skin cancer accounts for more than 80% and 50%, respectively, of all new cancers diagnosed each year [5, 6]. Surprisingly, within the United Kingdom (UK), where UV exposure in terms of both UV levels and hours of sunshine is significantly less, latest figures show skin cancer still accounts for more than 30% of new cancer diagnosed each year [1].

 Such alarming figures are partly attributable to the growth and popularity of the IT industry [7]. Despite the adverse health effects, IT appears to be gaining considerable popularity and for many “having a tan” is considered highly desirable by many young females, for reasons such as enhancing appearance [8–12]. Furthermore, specifically within the UK, where the weather is considerably mixed, using IT to achieve the desired tanned look is both a more affordable and widely available alternative to young people than travelling to warmer climates.

Primary prevention interventions have predominantly focused on the health consequences of such behaviour, and whilst they have been successful at enhancing knowledge and attitudes related to UV exposure and protection, few have been successful in modifying UV exposure/protection behaviours [13–17], and even fewer have focused directly on IT practices.

There is now a growing body of literature on interventions targeting the appearance consequences (e.g., premature skin aging—wrinkles and pigmented age spots) associated with UV exposure [10, 18–20]. The first systematic review assessing the efficacy of such interventions revealed that the synthesis of the findings was promising [21]. Reviewed studies that had assessed changes in UV exposure/protection intentions and behaviours revealed significant findings in favour of appearance-focused interventions. However, the interventions only had moderate success in modifying UV exposure behaviours. All studies were conducted within the USA and only three focused on IT behaviour. These types of interventions may be a successful alternative; however, the findings cannot be easily generalised to other countries of differing climates, for example, the UK, without knowing if appearance is a primary motive of this behaviour [21].

To aid our understanding of adherence and nonadherence to health-related behaviours, emphasis has been placed on theoretical models such as the theory of planned behaviour—TPB [22]. The basic principle of the TPB is that the immediate predictor of engagement in a volitional behaviour is *intention *and that an individual's intention is influenced by three principal determinants, attitude, subjective norm (SN), and perceived behavioural control (PBC) [22].

Few researchers have applied the TPB model to understand UV exposure and protection [23–26]. Their findings suggest that the model is a useful theoretical framework for understanding the motivations of such behaviour. However, even fewer studies have extended the model to include factors that have been at the forefront of research when referring to the motivations of tanning (e.g., appearance factors), none focus on a UK sample [27, 28].

Thus, whilst appearance-based interventions may be a breakthrough alternative to health consequence interventions, to date there is no comprehensive picture of the factors that influence the decisions to indoor tan within young people within the UK. To develop interventions that focus on appearance, further research is required to examine the factors that influence such decisions, in order to ensure that interventions are developed based on theoretical underpinnings of the behaviour. Thus, the aim of the study was to examine young people's IT intentions and behaviour living within the UK using an extended TPB model that included variables on “appearance reasons to tan,” “perceived susceptibility to damaging appearance”, “perceived susceptibility to health consequences”, and “tanning knowledge”.

## 2. Method

### 2.1. Sample and Procedure

Prior to data collection, ethical approval from the institutional research ethics committee was obtained. All students at the UK University College within the West Midlands were invited by email to take part in a prospective questionnaire survey on “young adults' views and behaviour towards IT”. Of the 284 who volunteered (approximately 10% of the entire student population), 210 young adults (44 male and 166 female mean age 21.37 ± 3.30 years), indicated that they had indoor tanned within 12 months (referred to as the tanner-T group); a usual cut-off used within the tanning literature. Whereas 71 young adults (23 males and 48 females, mean age 22.12 ± 4.05 years) reported not to have tanned within 12 months, thus referred to as the nontanners (NT) group. Three questionnaires were disregarded, due to incomplete responses. Participants firstly completed a self-report questionnaire by email (Time 1 or T1) that measured the constructs of the TPB, the proposed additional variables, and demographic characteristics. Two weeks later the sample were contacted by email and asked to report their IT behaviour preceding the two weeks (Time 2 or T2); responses constituted a 71% response rate; reminder emails were also sent out. As an incentive, participants had the opportunity to enter a prize draw to win gift vouchers. Sample size was determined as having sufficient power (0.8) to detect for a moderate effect size with the alpha set at 0.05 [29]. Minimum required sample size was 108.

### 2.2. Measures

#### 2.2.1. Demographic Characteristics

Basic demographic information was collected from all participants (Table 1). Skin type (e.g., tan and burn tendencies) was assessed according to method(s) published elsewhere [30]. For example, (I) I always sunburn, never tan, (II) usually sunburn, tan with difficulty, (III) sometimes mild sunburn, tan about average, (IV) rarely sunburn, tan with ease, (V) very rarely sunburn, natural brown skin, (VI) very rarely or never sunburn, natural black skin.

#### 2.2.2. Theory of Planned Behaviour Measures

T1 questionnaire included the traditional TPB constructs as well as the proposed additional variables. An IT behaviour item was measured at T2. The constructs of the TPB were developed in line with recommendations [31, 32], and several response items selected were similar to those used within the literature [25, 33]. Unless otherwise stated, all items were measured on seven-point response scales. All construct items were then aggregated to form a composite variable (e.g., PBC, attitude, SN, knowledge). A reliability analysis was conducted for all multiitem constructs/variables measured at baseline (T1) for the whole sample and the T and NT groups (Table 2). In line with recommendations from a recent discussion paper highlighting the problems with Cronbach's alpha as a measure of internal consistency, both Cronbach's alpha and Guttman's lambda 2 were reported [34].

#### 2.2.3. TPB Constructs

*Intention* was measured using one item; participants were asked to state how many times they intend to use an IT booth to obtain a tan in the next two weeks. *Attitude *towards IT was measured using eight bipolar adjective scales that tapped into both the instrumental and affective attitude concept (i.e., “for me, using an IT booth in the next two weeks to obtain a tan would be” harmful-beneficial/wise-foolish/unhealthy-healthy/good-bad/unpleasant-pleasant/enjoyable-unenjoyable/unrelaxing-relaxing/fun-boring). Interitem reliability of this scale was excellent. *SN* was measured using two items that tapped into the injunctive and descriptive concept, using a scale ranging from strongly disagree to strongly agree (i.e., “people in my social network, for example, family/friends, girlfriend/boyfriend, partner/spouse who are important to me would approve of me using IT booths in the next two weeks to obtain a tan”). Inter-item reliability of this scale was good. *PBC *was measured using four items that tapped into both the controllability and self-efficacy concept (i.e., “How confident are you that you could use an IT booth in the next two weeks to obtain a tan if you wanted to?” “not at all confident to very confident”). This scale had low inter-item reliability.

#### 2.2.4. Additional Variables

*Appearance reasons to tan* was measured using nine items from the “appearance motives to tan and not tan scale”—subscale “appearance reasons to tan” [35]. In order to fit the aim and purpose of this research slight wording modifications were made (e.g., reference made to IT) and the scale ranged from strongly disagree to strongly agree. Items consisted of statements, such as “having a tan gives me more sex appeal”, and “I tan because it makes me more attractive”. This scale had excellent inter-item reliability. *Perceived susceptibility to damaging appearance* was measured using nine items from the “appearance reasons to tan and not tan scale”—subscale “appearance reasons not to tan” [35]. Again slight modifications were made to the wording and items were measured on a scale ranging from strongly disagree to strongly agree. Items consisted of statements, such as “I'm concerned about getting blemished skin as a result of IT”, “I'm concerned about freckling from IT”. This scale also had excellent inter-item reliability. *Perceived susceptibility to health consequences* (e.g., skin cancer) was measured using three items on a scale ranging from strongly disagree to strongly agree (i.e., “I am concerned if I use IT booths frequently I will enhance my chances of skin cancer”). Again, this scale had excellent inter-item reliability. *Tanning knowledge* consisted of 11 items based on the effects of IT, UV safety, tanning fashion trends, and factual tanning statistics from websites on skin cancer [1, 3, 36]. Items consisted of statements with a mixture of closed-ended and open-ended response options, such as “IT booths cannot cause skin cancer”, with response options of true/false/do not know or open-ended items, such as “What is the minimum age you must be by law to use an IT booth?”, with answers scored 1 for correct, 0 for incorrect or do not know. The interitem reliability of this scale was determined to be only moderate; however low/moderate internal consistency does not necessarily mean that the scale items are not a composite measure of the variable of interest [37]. Tanning knowledge can be seen as multidimensional (e.g., as there are different dimensions to it—knowledge of UV consequences, knowledge of protective behaviours), in that whilst some of the respondents may have good knowledge of how to protect themselves against UV rays, they may be less knowledgeable about the consequences of exposure to UV rays.

*IT behaviour* was measured using a single open-ended item, “How many times did you use an IT booth in the previous two weeks?”

### 2.3. Statistical Analysis

Initial equivalence tests were conducted on the data obtained from those who had been tanners (e.g., T group) and those who reported not to have used IT booths in the previous 12 months, nontanners, (e.g., NT group), descriptive and simple statistical analysis procedures (e.g., independent *t*-tests, and chi-squares) were performed on the demographic and the extended TPB questionnaire constructs/variables. Welch's *t* was reported instead of the more conventional independent *t*-value when the variances of the two groups differed by more than twice as the sample sizes were unequal [38]. Due to the violation of the chi-square, “expected cell frequency” assumption, not all chi-square tests could be conducted on the categorical variables.

The primary analyses were conducted using two hierarchical forced-entry regression analyses procedure; this method is ideal when the predictors (i.e., IVs) are selected based on previous research and when the researcher knows something about the predictability of some of the predictors; thus the model allows the researcher to enter IVs into the model in a specific order [38]. Multiple regression assumptions were considered. All analyses were conducted using the Statistical software—SPSS version 19.

## 3. Results

### 3.1. Description of Sample and Baseline Equivalence of the Groups

Table 2 depicts T1 characteristics of the two groups (e.g., T versus NT) and where possible, statistical analyses were carried out to assess for significant differences. The ethnic composition of the whole sample was predominantly white origin (85%). None of the sample reported a personal diagnosis of skin cancer; the T group revealed a greater percentage of family members being diagnosed with skin cancer than the NT group. Skin type distribution revealed that a greater percentage of the T group reported skin type III (43%, *n* = 90), whereas 54% (*n* = 38) of the NT group reported skin type IV or V. The skin type distribution for the T group was consistent with previous results for this population [20].

As expected, significant differences for the TPB measures and the additional variables between the T and NT group emerged. Significant differences were found for all but one variable, “perceived susceptibility to damaging appearance”. The T group revealed stronger positive perceptions towards IT than the NT group on attitude, SN, PBC, “appearance reasons to tan”, “tanning knowledge”, and, of course, greater intentions. The NT group revealed greater perceived susceptibility to health consequences than the T group (mean of 5.1 and 4.2 resp., *P* < 0.01).

### 3.2. Multivariate Analyses

To identify the prominent predictors of intention and behaviour to the use IT booths, two hierarchical forced-entry regression analyses were used to analyse the T1 and T2 data. IT intention was regressed on the TPB constructs in block one of the regression equation, and block two consisted of the additional variables; “appearance reasons to tan”, “perceived susceptibility to damaging appearance”, “perceived susceptibility to health consequences”, and “tanning knowledge”. 

IT behaviour was regressed on intention and PBC in block one, and block two consisted of the TPB and the additional variables. 

The correlation matrix (Table 3) demonstrated the relationship between the variables. 

As depicted in Table 4, the TPB model (PBC, attitude, SN) made a significant contribution (8%, *P* < 0.001) towards explaining IT *intentions*, with *PBC *and *attitude* making a significant unique contribution (*P* < 0.01 and *P* < 0.05 resp.). Entry of the additional variables in block two explained a further 9% of the variance in IT *intentions*, with both *PBC *and the variable *appearance reasons to tan *making a significant contribution (*P* < 0.05 and *P* < 0.001 resp.). Overall, the extended TPB explained 17% of the variance in IT *intention*.

IT *behaviour *was first regressed on *intention* and *PBC*. The model accounted for 70% of the variance in behaviour (*P* < 0.001), with intention making a significant unique contribution (*P* < 0.001). Entry of the additional variables accounted for a further 1% in block two, with the variables *intention *and *appearance reasons to tan *making a unique significant contribution (*P* < 0.001 and *P* < 0.05 resp.). Overall, the extended TPB explained 71% of the variance in IT *behaviour.*

## 4. Discussion

The findings derived from the data were interesting on many levels. A reassuring finding was that the tanning group reported using IT booths an average of 5 times in the last year, whereas student populations had been found to indoor tan up to 37 times per year [20]. Furthermore, over a two-week period the tanning group reported using an IT booth an average of 1.1 times. Other encouraging findings were that on average the tanning group had less favourable attitudes towards IT. Both family and friends were likely to disapprove of this behaviour and their perceived susceptibility to damaging appearance and health consequence was relatively high. Knowledge and PBC was also high; however the latter two outcomes should be interpreted with caution due to moderate/low inter-item reliability.

Consistent with the limited research on the TPB and IT, the TPB model was successful in predicting behavioural intentions. However, the variance explained by the TPB model on IT was relatively small compared to the previous research [25, 27]. The TPB model (PBC attitude and SN) accounted for 8% of the variance in IT intentions, with only PBC emerging as a significant unique predictor. This indicates that individual attitude and SN were not making a unique significant contribution to IT intentions.

When the model was extended to include the additional variables, a further 9% of the variance was explained with PBC and the variable “appearance reasons to tan”, both emerging as significant unique predictors, and the latter variable making a greater significant contribution. The magnitude of the latter variable was contrary to expectations, implying that the “appearance reasons to tan” outweighs two of the main constructs of the TPB, one's attitudes towards the behaviour and friends and family perceptions towards IT.

IT behaviour was regressed upon intention and PBC in the first instance. The model predicted 70% of the variance in IT behaviour, with intention making a significant unique contribution.

Overall, the findings suggest that the variable “appearance reason to tan” is the strongest predictor towards intention, and intention is the strongest predictor towards behaviour with “appearance reason to tan” a close second.

The emphasis of appearance being the primary motivation to IT is consistent with a number of authors who have been advocating the need for appearance-focused interventions to reduce UV exposure, as evidenced in a recent systematic review [21]. The findings from the systematic review showed the power of the “appearance” variable. However, the impact of this variable was not assessed on a UK sample; thus it was not clear whether this variable was a primary predictor of this behaviour within the UK. 

Thus considering the findings found from this study the authors now believe that appearance interventions are justified within a UK young adult population. However, further consideration will need to be given to how appearance perceptions can be altered, considering the importance of this to young adults' daily lives. Encouraging other alternative healthy approaches (e.g., diet, exercise, and clothing) to address appearance enhancement has been put forth [9]; even sunless tanning lotion may be a more viable alternative. The authors feel that an in-depth exploration of the factors associated with appearance enhancement within young adults is also warranted and such findings would help to shape appearance-focused interventions. 

Considering the baseline data of the two different groups (e.g., tanners—T and nontanners—NT), no unexpected findings were revealed. The T group had stronger positive perceptions towards IT than the NT group on attitude, SN, PBC, and “appearance reasons to tan”. Although, the T group revealed greater tanning knowledge and of course greater intentions, the former is not out of the ordinary as research has shown that tanners are knowledgeable about this behaviour. The NT group revealed greater “perceived susceptibility to health consequences” than the T group. It may be interesting to also explore what motivates appearance enhancement of NT and the alternative methods they use to address appearance.

The findings of the study should be considered within the context of its limitations. A convenience sample was selected, which is associated with selection bias, with more of the volunteers being tanners or at least interested in IT than the wider population.

The sample was also young adults (students) from one University College; this population was specifically chosen as young people are frequent IT users and students have been reported to engage in a number of unhealthy practices [39]. This may impact upon the generalisability of the findings to other higher education (HE) institutions or nonstudent populations. Therefore, as there is limited research using an extended TPB on IT, and more specifically this is the first study to do so within the UK, a replication of the findings in other HE institutions and nonstudent populations would provide insight into how representative the findings are. It is also important to note that a larger percentage of females participated in the study than males; thus the findings may be more representative to females. 

Behaviour was also measured by self-report, an objective measure such as “change in skin colour” using skin reflectance spectrophotometer equipment; whilst expensive, it would enhance the validity of the findings. Furthermore, it is important to note, while not unique to this paper, intention was only assessed using a single item. Data collection was also conducted during a short time frame (2 weeks) and only in one season. Finally, response rate must also be taken into consideration—response rate for Time 2 (T2) was only 71% (i.e., <80%).

In conclusion, an extended TPB model successfully predicted IT intentions and behaviour. Notably, the findings highlighted the power of a tanned appearance for appearance enhancement in young adults within the UK and how at this current time in their lives this outweighs any appearance/health consequences. Thus, the findings have important clinical implications. It is not unreasonable at this stage to conjecture that appearance-focused interventions may be a promising method to reduce UV exposure within a UK sample. As this is the first study to assess an extended model of the TPB (which directly includes a measure of appearance reasons to tan) on IT in a UK young adult population, the findings are insightful, and the authors feel that interventions should now be developed to mirror such findings.

## Figures and Tables

**Table 1:** Characteristics of tanners (T; *n* = 210) versus nontanners (NT; *n* = 71).

Characteristics	T	NT	Difference
Age mean (SD)	21.4/3.3	22.1/4.1	*t*_(279)_ = 1.571,
			eta squared = .01
Sex *n* (%)			*χ*_(1)_^2^ = 3.826*, phi = −.117
Male	44/21.0	23/32.4	
Female	166/79.0	48/67.6	
Ethnicity *n* (%)			
White	204/97.1	34/47.9	
Other	6/2.9	37/52.1	
Skin type *n* (%)			
I	12/5.7	4/5.6	
II	45/21.4	9/12.7	
III	90/42.9	17/23.9	
IV	52/24.8	19/26.8	
V	8/3.8	19/26.8	
VI	3/1.4	3/4.2	
Average IT use past yr. mean (SD)	5.35/9.96	.00/.00	*t*_(209.000)_ = 4.518***,
			Eta Squared = .09
Age first used ITB mean (SD)	17.75/208	18.00/1.79	*t*_(213)_ = −.290,
			Eta Squared = .00
TPB and additional constructs mean (SD)			
Attitude	3.4/1.2	2.3/1.2	*t*_(279)_ = 6.573***,
			Eta Squared = .13
SN	3.3/1.6	2.1/ 1.4	*t*_(279)_ = 5.999***,
			Eta Squared = .11
PBC	5.2/.80	4.7/1.1	*t*_(94.480)_ = 3.816***,
			Eta Squared = .13
Intention	.5/.9	0	*t*_(209.000)_ = 8.771***,
			Eta Squared = .27
Appearance reasons to tan	5.3/2.0	2.8/1.8	*t*_(279)_ = 11.713***,
			Eta Squared = .33
Damaging appearance	5.0/1.4	4.8/1.8	*t*_(279)_ = 1.007,
			Eta Squared = .00
Health consequences	4.2/2.0	5.1/2.2	*t*_(279)_ = 3.341**,
			Eta Squared = .04
Tanning knowledge	8.4/1.8	7.7/2.1	*t*_(279)_ = 3.005**,
			Eta Squared = .03

*P* ≤ 0.05*, *P* < 0.01**, *P* < 0.001***.

**Table 2:** Reliability analysis of the multiitem measures by sample group.

Factors (items)	Whole sample at T1	T	NT
TPB (alpha/lambda 2)			
PBC	.386/.409	.250/.299	.492/.526
Attitude	.893/.897	.887/.892	.859/.866
SN	.764/.764	.752/.752	.667/.667
Additional constructs			
Appearance reasons to tan	.983/.983	.976/.976	.972/.973
Perceived susceptibility to:			
Damaging appearance	.924/.929	.916/.921	.940/.945
Health consequence	.951/.952	.947/.948	.964/.964
Tanning knowledge	.485/.509	.393/.437	.608/.629

**Table 3:** Zero-order correlations between the extended TPB variables.

	1	2	3	4	5	6	7	8	9
(1) Behaviour		.836***	.205**	.184*	.086	.408***	−.028	−.039	.047
(2) Intention			.251***	.177**	.075	.393***	−.040	−.019	.085
(3) PBC				.327***	.354***	.263***	.055	.064	.253
(4) Attitude					.371***	.198**	−.201**	−.123*	−.016
(5) SN						.158*	.051	.169**	.251***
(6) Appearance reasons to tan							−.101	−.127*	.159*
Perceived susceptibility to:									
(7) Damaging appearance								.335***	.133*
(8) Health consequence									.094
(9) Tanning knowledge									

*P* ≤ 0.05*, *P* < 0.01**, *P* < 0.001***.

**Table 4:** Hierarchical regressions of the predictors of IT intention and behaviour.

Predictor						CI 95% for *B*	
	*R* ^2^	Δ*R* ^2^	*F*	Beta	*t*	Lower B	Upper B
*Intentions *							
(1)	.079	.069	7.905***				
PBC				.210	3.325**	.075	.294
Attitude				.152	2.340*	.015	.180
SN				−.030	−.453	−.081	.051
(2)	.166	.144	7.755***				
PBC				.132	2.051*	.005	.227
Attitude				.074	1.116	.036	.131
SN				−.065	−.983	−.098	.033
Appearance reasons to tan				.330	5.240***	.086	.190
Perceived susceptibility to:							
Damaging appearance				−.023	−.385	.078	.052
Health consequence				.013	.215	.042	.053
Tanning knowledge				.007	.110	.052	.058
*Behaviour *							
(1)	.702	.695	115.117***				
Intention				.834	20.560***	.815	.988
PBC				−.048	−.529	−.119	.069
(2)	.710	.698	58.891***				
Intention				.830	18.654***	.776	.959
PBC				−.031	−.682	−.130	.063
Attitude				.029	.630	−.049	.094
SN				.021	.452	−.041	.066
Appearance reasons to tan				.098	2.229*	.006	.106
Perceived susceptibility to:							
Damaging appearance				.030	.705	−.036	.076
Health consequence				−.015	−.360	−.048	.033
Tanning knowledge				−.037	.874	−.066	.025

*P* ≤ 0.05*, *P* < 0.01**, *P* < 0.001***.
